# Review of Thin Film Nanocomposite Membranes and Their Applications in Desalination

**DOI:** 10.3389/fchem.2022.781372

**Published:** 2022-01-28

**Authors:** Jegatha Nambi Krishnan, Kaarthick Raaja Venkatachalam, Oindrila Ghosh, Krutarth Jhaveri, Advait Palakodeti, Nikhil Nair

**Affiliations:** ^1^ Department of Chemical Engineering, Birla Institute of Technology and Science Pilani, K.K. Birla Goa Campus, Zuarinagar, India; ^2^ Strategic Engagement and Analysis Group, Rocky Mountain Institute, Boulder, CO, United States; ^3^ Process and Environmental Technology Lab, Department of Chemical Engineering, KU Leuven, Leuven, Belgium

**Keywords:** reverse osmosis, thin film nanocomposite membrane, desalination, interfacial polymerization, chlorine resistance, antifouling, permeability

## Abstract

All over the world, almost one billion people live in regions where water is scarce. It is also estimated that by 2035, almost 3.5 billion people will be experiencing water scarcity. Hence, there is a need for water based technologies. In separation processes, membrane based technologies have been a popular choice due to its advantages over other techniques. In recent decades, sustained research in the field of membrane technology has seen a remarkable surge in the development of membrane technology, particularly because of reduction of energy footprints and cost. One such development is the inclusion of nanoparticles in thin film composite membranes, commonly referred to as Thin Film Nanocomposite Membranes (TFN). This review covers the development, characteristics, advantages, and applications of TFN technology since its introduction in 2007 by Hoek. After a brief overview on the existing membrane technology, this review discusses TFN membranes. This discussion includes TFN membrane synthesis, characterization, and enhanced properties due to the incorporation of nanoparticles. An attempt is made to summarize the various nanoparticles used for preparing TFNs and the effects they have on membrane performance towards desalination. The improvement in membrane performance is generally observed in properties such as permeability, selectivity, chlorine stability, and antifouling. Subsequently, the application of TFNs in Reverse Osmosis (RO) alongside other desalination alternatives like Multiple Effect Flash evaporator and Multi-Stage Flash distillation is covered.

## 1 Introduction

The United Nations World Water Development Report 2020: Water and Climate Change states that in accordance with a study conducted by the 2030 Water Resources Group (WRG), the world will have only 60% of the water it needs by 2030, if it continues on its current trajectory (UNESCO, 2020). This scarcity has risen out of the ever growing gap between the overwhelming demand, through agriculture, industries, urbanization, rapidly rising population; and the worryingly low supply of consumable water. This is where the importance of recycling water and converting the abundantly available seawater to usable form by the process of desalination becomes critical. Over the previous decade, membrane based technologies have developed significantly (The World Bank, 2007; Lind et al., 2009a; Li et al., 2013) and the growth in total desalination capacity across the world has been staggering. Among the different desalination techniques such as electrodialysis, Mechanical Vapor Compression (MVC) and Nano-Filtration (NF), Reverse Osmosis (RO) is the most popularly used technique. Energy consumption of RO membranes in 2013 was 1.8 k Wh, which makes the process much less energy intensive than the other available options, such as MSF (multi-stage flash distillation) and MEF (multiple effect flash distillation) (Buonomenna, 2013). Over 60% of today’s desalinated water comes through RO technology.

For the purpose of desalination and other similar applications, various kinds of membranes are fabricated like thin film composite membranes (TFCs) and thin film nanocomposite membranes (TFNs). TFNs are a modification of the existing TFCs prepared through interfacial polymerization (IP). The modification is in the form of nanoparticles being incorporated into a thin polyamide (PA) dense layer at the top of the TFC membrane, aimed at improving its performance (Jeong et al., 2007). This enhancement could be varied such as in the form of improved water permeability and solute rejection.

The TFN membrane was introduced by Hoek (Jeong et al., 2007) in which TFNs were synthesized by embedding zeolite NaA nanoparticles (0.004–0.4% w/v) in the PA layer. The PA layer of the composite membrane was made of m-phenyldiamine (MPD) and trimesoyl chloride (TMC). This new concept showed a significant enhancement in the membrane flux while maintaining a comparable solute rejection to the traditionally prepared TFC membrane. This improvement in permeability due to the super-hydrophilic molecular sieve nanoparticle pores which provide distinct channels for the flow is ascribed (Jeong et al., 2007).The following section discusses the evolution of membrane technology from its first instance of application in a water separation process in 1748 to the currently produced more advanced TFNs.

## 2 Background and History

**FIGURE 1:**
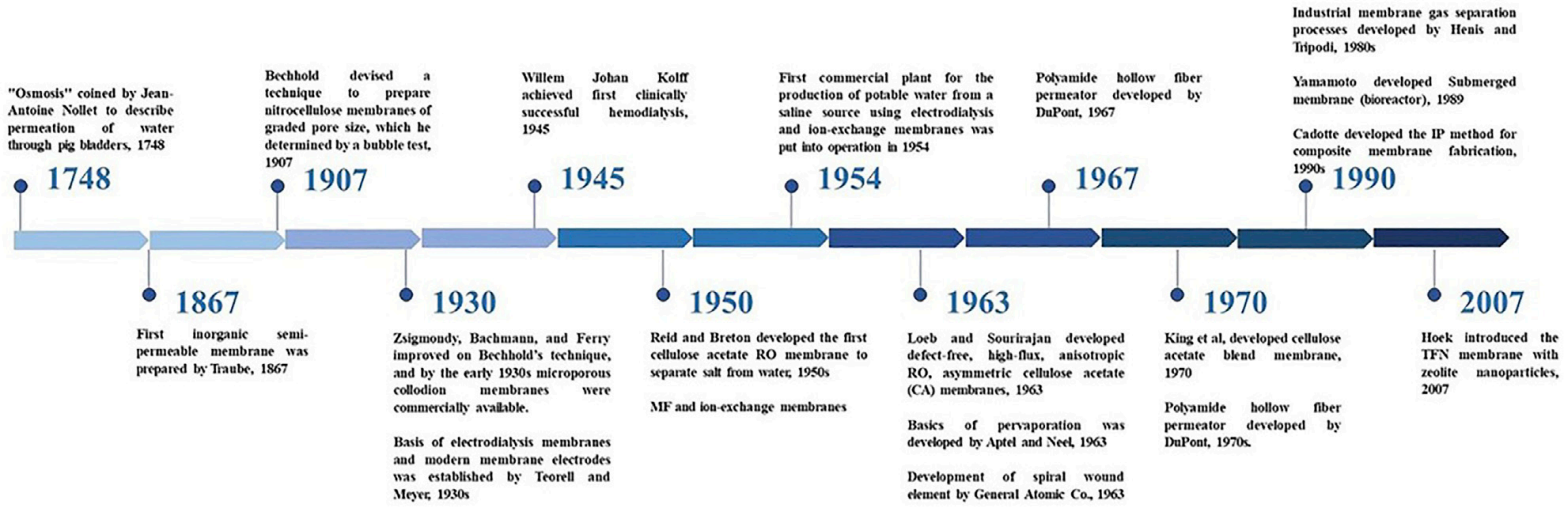
History of the development of membrane technology.

The application of membranes for separation of small solute particles from water was first reported in 1748 by Jean-Antoine Nollet (Williams, 2003). However, it was only in the 1850s that Traube and Pfeffer became the first to study osmosis using ceramic membranes. In 1959, the first cellulose acetate RO membrane capable of separating salt from water was developed by C. E. Reid and E. J. Breton (Williams, 2003). These membranes had a very low flux due to the excess thickness a free standing membrane was required. It was in 1962 that a major advancement was made by Loeb and Sourirajan, who developed an anisotropic cellulose acetate membrane. It had a thin layer on top of a highly porous and thick substrate (Williams, 2003). This made RO a practical desalting process exhibiting salt rejection values of about 99.5%. Subsequently, different types of designs such as tubular, hollow fiber, and spiral wound were developed as shown in Figure 1 to utilize the membranes on a commercial scale (Li and Wang, 2013).

The Loeb-Sourirajan membrane became an industry standard until the 1970s, when the IP method for composite membrane fabrication was developed by John Cadotte of Dow FilmTec (Li and Wang, 2013). This involved placing an ultra-thin aromatic top layer on a porous ultrafiltration membrane via the IP process. The aromatic structure made it highly durable making it the technologically most advanced membrane at that time. The applications of these membranes have been extended to the removal of other dissolved solutes from various feed waters (Ex. FT-30 membrane developed by Cadotte at Dow FilmTec). Modifications in membrane design, configuration, and introduction of pre-treatment and post-processes allowed the energy consumption of RO desalination to be reduced from six k Wh/m^3^ (in the 1980s) to 1.8 k Wh/m^3^ (Li and Wang, 2013). The FilmTec membranes continued to dominate the US market till Hoek introduced the first TFN membrane for brackish water reverse osmosis (BWRO) membranes in 2007. In that work, the membrane had zeolite nanoparticles dispersed in the organic solution of an interfacial polymerization reaction. This was commercialized in 2011 through a start-up called NanoH_2_O (NanoH2O and Inc. InterNano, 2021). Since then, TFNs have been at the center of attention in membrane technology. The following section delves deeper into the structure and synthesis of TFCs along with its limitations that make TFNs a better choice in most applications.

## 3 Thin Film Composite Membranes

TFC membranes are the precursors to TFNs. Composite membranes are a type of asymmetrical membranes that have a dense top layer and a porous support made of different materials. Commercial RO membranes were initially obtained from two kinds of membranes: Polyamide (PA) and Cellulose Acetate (CA) (Li and Wang, 2010; Rana T. M. D. et al., 2011). PA TFCs have two layers; a porous substrate layer (usually made of polysulfone) and a thin layer made of polyamide formed on it (Li and Wang, 2010). The top layer is concerned with permeation properties of the membrane, while the sub-layer, which is porous, provides mechanical strength and support. The advantage of having the two layers made of different chemicals is that each layer can be individually synthesized or customized so as to optimize the overall performance of the membrane (Louie et al., 2006).Compared to CA membranes, TFCs exhibit better salt rejection, water flux and resistance to biological attacks, apart from being able to operate at wider range of pH (varying from 1–11) and temperatures (varying from 0–45°C) (Li and Wang, 2010). The following subsection discusses the techniques involved in the fabrication of TFCs.

### 3.1 Synthesis of TFCs

The methods of fabrication include techniques such as IP, solution mixing and polymer melt blending (Kim and Deng, 2011) of which IP has been the most common since its introduction in 2007 (Jeong et al., 2007). Generally an asymmetric membrane prepared through phase inversion is the support layer (Ghosh and Hoek, 2009). Polymers generally used to form the support layers are polysulfone (PSF), polyimide (PI), polyethersulfone (PES), polyacrylonitrile (PAN), and polypropylene (PP) (Ghosh and Hoek, 2009). Various techniques can be used to deposit a thin layer on a porous sub-layer one of these is IP. The traditional synthesis technique requires the dissolution of a difunctional amine in water and a trifunctional acid halide in an organic solvent. The solvent is usually chosen such that water and the solvent are immiscible. The support membrane (usually PSF) is initially immersed in the aqueous solution and, after saturation, is then immersed into the organic phase. Upon immersing in the organic phase, there is a polymerization reaction between the monomers to form the thin layer (Ghosh and Hoek, 2009). Alternatively, there is another method of IP known as the SIM method. In this method, the organic phase is poured over the membrane as it already has amines present on it due to phase inversion. This method is more efficient as there is more complete wetting, which results in a more homogenous, defect-free layer as compared to the traditional methods (Hermans et al., 2014).

TFCs have been a boon to membrane based technologies. However, they do have some limitations. These have been discussed in sub Section 3.2.

### 3.2 Limitations of TFCs

One of the biggest advantages of a TFC is that one can control and optimize each layer individually to improve its function. A high solvent flux can be obtained without compromising on the salt rejection. In addition, they are also much stronger and more stable (Petersen, 1993). However, in spite of the above advantages, TFCs have a few limitations which cannot be neglected.

One of the main drawbacks is its low resistance to chlorine (Lau et al., 2012). Chlorination of the main group present in the PA layer increases the hydrophobic nature of the membrane, which in turn decreases flux (Hermans et al., 2015). Chlorine is abundantly present in waters to be treated as chlorination is an important disinfection and pre-treatment step. Therefore, this is a significant performance inhibitor. TFCs are also very susceptible to fouling over time by microorganisms or organic compounds which leads to decline or deterioration of membrane performance. The process of de-fouling increases costs and energy consumption. In addition, high temperatures can also cause collapsing or compactness of pores in the membrane (Hermans et al., 2015).

To overcome these limitations, various methods were developed with the intent to improve the fouling resistance and chlorine resistance of TFCs thereby increasing their performance. This can primarily be done through modifications in the substrate. The following subsection discusses this aspect in details.

### 3.3 Methods of Membrane Modification

#### 3.3.1 Modifications in the Substrate

Polysulfones are very commonly used as a substrate for the fabrication of TFCs. In recent years, substantial research work has been conducted with the aim to enhancemembrane performance by modifying the substrate layer, either by the inclusion of organic solvents such as n-methyl pyrolidone in precipitation, or by addition of hydrophilic agents such as polyethylene glycol (Zhou et al., 2009). Methods that have been reported to increase the fouling resistance and chlorine stability have been discussed in the subsequent sub sections.

#### 3.3.2 Increase in the Fouling Resistance

The fouling of the membrane can lead to decreased water flux, which in turn leads to increased energy consumption to pump water through membranes. TFCs can be modified by physical or chemical methods so as to reduce fouling (Li and Wang, 2010). Physical methods include applying a coating on the surface of the membrane without interfering with the chemical structure of the material. Polyvinyl alcohol and polyethyleneimine are some of the polymers that have been used in this manner in recent studies (Kim et al., 2004; Louie et al., 2006; Zhou et al., 2009). As an example, polyethyleneimine can invert surface charges on polyamide membranes so as to reduce fouling by cationic substances by electrostatic repulsion (Zhou et al., 2009).

Chemical modification methods can also be used to increase fouling resistance. Monomers can be covalently attached to the monomers on membrane surfaces by radiation or redox grafting methods. This modification of the surface of membranes can lead to a decrease in contaminant adsorption for certain polymers (Li and Wang, 2010).

#### 3.3.3 Increase in Chlorine Stability

The chlorine resistance of the PA layer of the membranes depends upon the chemical nature of the diamine structure. Chlorine resistance will be high if the amino groups have the following structures: i)aromatic diamines that have a mono methyl or chlorine substitutes at the ortho position of the amino groups; or with amino groups at ortho-position, compared with those at meta- and para-position; ii) aliphatic or cycloaliphatic diamines that possess a secondary amino group or a short methylene chain length between end amino groups; iii) secondary aromatic diamines (Li and Wang, 2010).

Chlorine resistance can also be increased by the addition of certain types of monomers. For example, membranes produced via IP by introducing–OH functional group containing monomers such as m-aminophenol and bisphenol-A show greater chlorine resistance than MPD -TMC polyamide membranes (Li and Wang, 2010). Another method is incorporating ester linkages by substituting aromatic amines, as this reduces the number of available sites for chlorine attack (Li and Wang, 2010).

Incorporation of nanoparticles in TFCs results in TFNs. These have a large number of preferable characteristics. The details of TFNs have been discussed in Section 4 starting with the processes developed for the synthesis of the same.

## 4 Synthesis of TFNs

**FIGURE 2:**
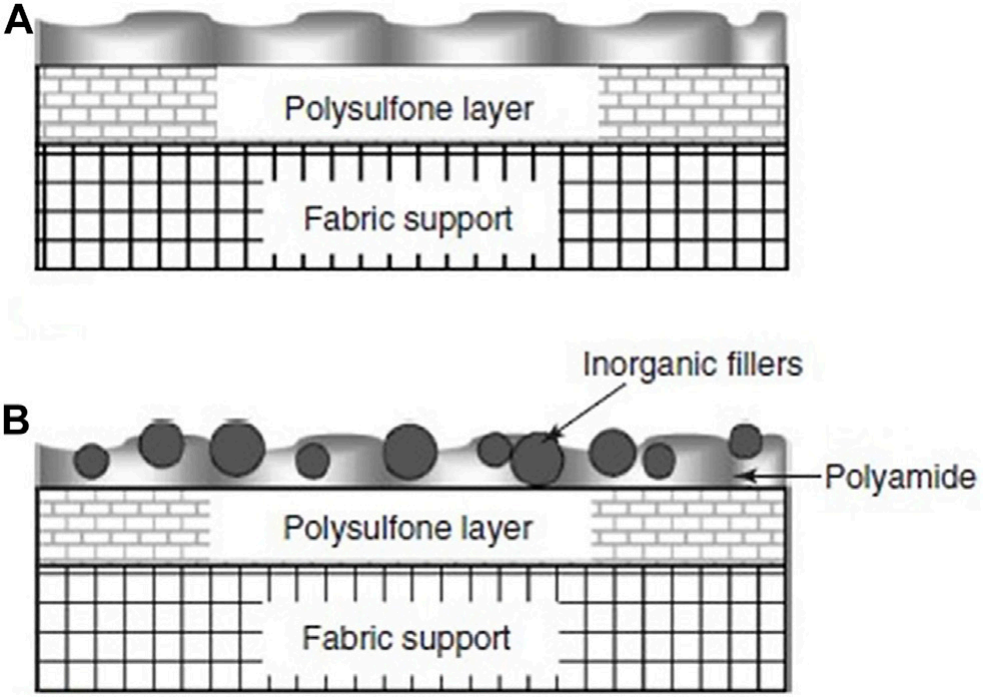
Conceptual illustration of PA **(A)** TFC and **(B)** TFN membrane structures (Zhou et al., 2009).

TFNs can be synthesized by IP, solution mixing or polymer melt blending (Kim and Deng, 2011). One of the more commonly used techniques is IP (Jeong et al., 2007). Preparation of TFNs takes place in almost the same manner as TFCs, except for a step which involves the addition of fillers. Figure 2 illustrates PA in both TFC and TFN membrane structures (Li and Wang, 2010).

**FIGURE 3:**
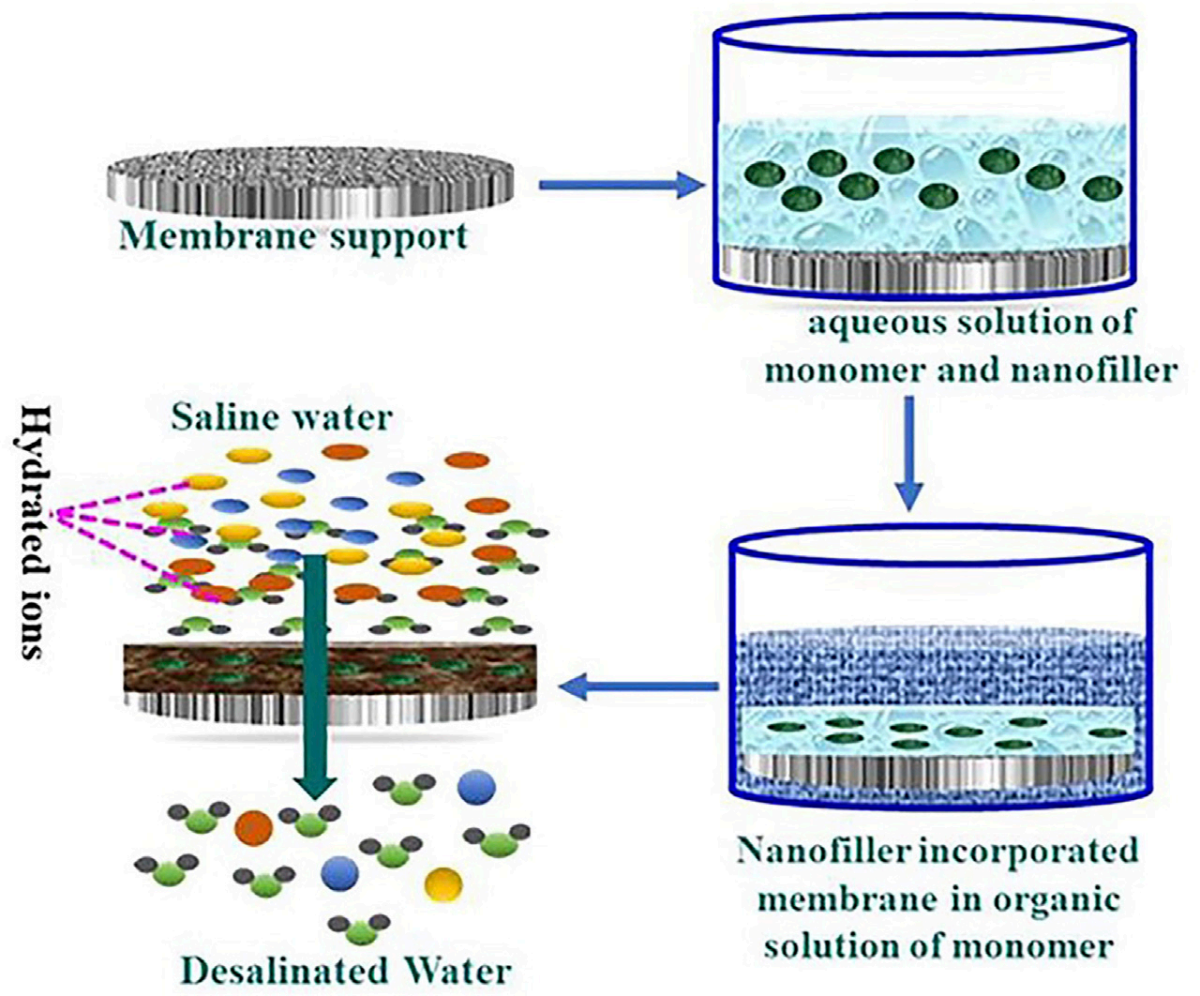
Diagrammatic representation of manufacturing TFN membrane through interfacial polymerization in the presence of nanofillers (Kumar et al., 2020).

**FIGURE 4:**
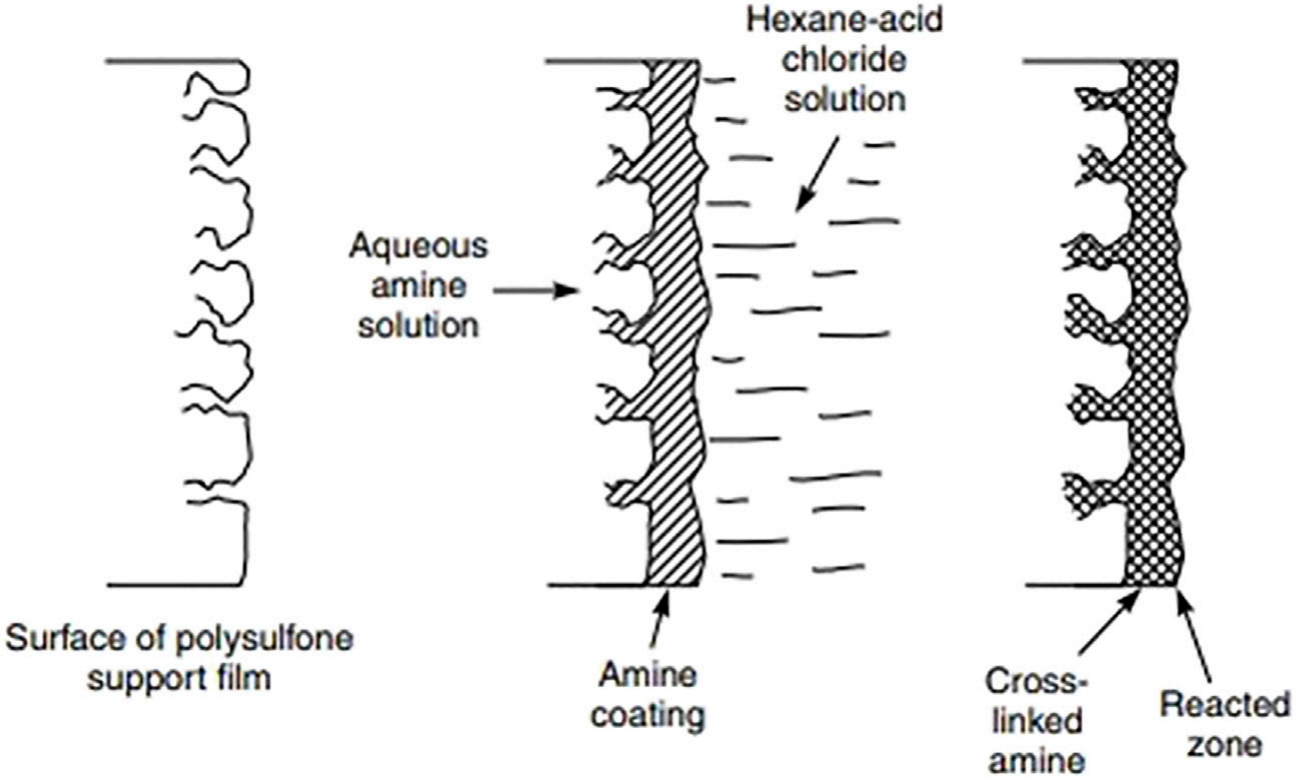
Schematic of the interfacial polymerization process (Kim and Deng, 2011).

The fillers can be added to aqueous or organic phase (Figure 3) based on the properties of the nanoparticle fillers used. The membranes can be dipped into a nanoparticle solution (Shenvi et al., 2015). Once the nanoparticles are embedded in one of the phases, IP is carried out as per standard procedure (Figure 4). In IP, an aqueous amine solution is first used to impregnate a microporous film. This is followed by a treatment using a multivalent cross linking agent that has been dissolved in a organic fluid, that is immiscible with water, for example, hexane. As a result, a thin polymer film is obtained at the interface of the two solutions. Table 1 summarizes the methods of synthesis and the important properties of various TFNs reported in literature (Petersen, 1993; Kim et al., 2004; Louie et al., 2006; Ghosh and Hoek, 2009; Jadav and Singh, 2009; Zhou et al., 2009; Park K. T. et al., 2010; Li and Wang, 2010; Fathizadeh et al., 2011; Kim and Deng, 2011; Rana T. M. D. et al., 2011; Zhang et al., 2011; Lau et al., 2012; Huang et al., 2013a; Kim E. S. et al., 2013; Alam et al., 2013; Baroña et al., 2013; Chan et al., 2013; Pendergast et al., 2013; Shen et al., 2013; Hermans et al., 2014; Zhao et al., 2014; Ghanbari et al., 2015; Hermans et al., 2015; Safarpour et al., 2015; Shenvi et al., 2015).

**TABLE 1:** Synthesis and properties of TFNs (Petersen, 1993; Kim et al., 2004; Louie et al., 2006; Ghosh and Hoek, 2009; Jadav and Singh, 2009; Zhou et al., 2009; Park K. T. et al., 2010; Li and Wang, 2010; Fathizadeh et al., 2011; Kim and Deng, 2011; Rana T. M. D. et al., 2011; Zhang et al., 2011; Lau et al., 2012; Huang et al., 2013a; Kim E. S. et al., 2013; Alam et al., 2013; Baroña et al., 2013; Chan et al., 2013; Pendergast et al., 2013; Shen et al., 2013; Hermans et al., 2014; Zhao et al., 2014; Ghanbari et al., 2015; Hermans et al., 2015; Safarpour et al., 2015; Shenvi et al., 2015).

Polymer matrix	Nanoparticle	Synthesis method	Properties	References
Polyamide(MPD—TMC)	Zeolite A (NaA) (50–150 nm)	IP with zeolite LTA in TMC hexane	Smoother, more hydrophillic, higher water permeability with equivalent salt rejection	Li and Wang, (2010)
Polyamide(MPD—TMC)	Zeolite A (NaA) (100,200,300 nm)	IP with aeolite LTA in TMC-isoparaffin	More permeable, negatively charged, thicker than PA TFC films	Li and Wang, (2010)
Polyamide(MPD—TMC)	Zeolite A(NaA or AgA) (140 nm)	IP with zeolite A in TMC isoparaffin	High water permeability, smooth interface, limited bactericidal activity for AgA membranes	Li and Wang, (2010)
Polyamide(MPD—TMC)	Zeolite A(NaA) 250 nm	IP with zeolite A in TMC-isoparaffin	Different post treatmentchanging the molecular structure, commercially relevant RO separation	Li and Wang, (2010)
Polyamide(MPD—TMC)	Commercial Silica nanoparticles LUDOX HS-40(16 nm) and TEOS hydrolyzedsilican(3 nm)	IP with adding silican in MPD aqueous solution	Tunable pore radius, increasing number of pores, higher thermal stability, high water flux and low salt rejection	Li and Wang, (2010)
Polyamide (MPD-BTC)	Commercial silver nanoparticles (50–100 nm)	IP with adding silver in BTC-HCFC	Slightly lower flux and higher rejection, higher antibiofouling effect	Li and Wang, (2010)
Polyamide(MPD—TMC)	Synthesized TiO_2_ (≤ 10 nm)	Self assembly of TiO_2_ on the neat MPD-TMC TFC surface	Higher salt rejection and lower flux, higher photocatalyticbatericidal efficiency under UV light	Li and Wang, (2010)
Polyamide(MPD—TMC)	Commercial TiO_2_ (30 nm)	IP with adding TiO_2_ in TMC-HCFC	Enhanced surface hydrophilicity, comparable water flux and higher salt rejection with limited amount of TiO_2_	Li and Wang, (2010)
Polyamide(MPD—TMC)	Commercial MWCNT(dia:9–12 nm; length: 10–15 um)	IP with adding MWCNTs in MPD aq. solution	Slightly lower salt rejection and flux; improved chlorine resistance with increase in MWCNT loading	Zhang et al. (2011)
Polyamide(MPD—TMC)	250 nm Linde Type-A zeolite	IP with adding Zeolite A in aq. solution of MPD, TEA, CSA, SLS, IPA	(1) Smoother, more hydrophilic surfaces (2) higher water permeability and salt rejection, and (3) improved resistance to physical compaction	Pendergast et al. (2013)
Sulfonated poly(arylene ether sulfone)—polyamide (MPD-TMC) copolypmer	Synthesized mesoporous silica nanoparticles (100 nm)	Interfacial polymerization with adding SiO_2_ in TMC-cyclohexane	High water flux and similar salt rejection	Park K. T. et al. (2010)
Polyamide(MPD—TMC)	Polyamide single walled aluminosilicate nanotubes	Ip followed by reacting different imogolite concentrations in 0.01% (w/v) TMC-hexane solution with the top surface of the MPD-soaked membrane	The hydrophilicity was increased as observed in the enhancement in water flux and pure water permeance, due to the presence of hydrophilic nanotubes. With the incorporation of the single-walled aluminosilicate nanotubes, higher permeate flux was achieved while sustaining high rejection of monovalent and divalent ions	Baroña et al. (2013)
Polyamide(MPD—TMC)	NanozeoliteNaX	IP over PES support. Immersed in aq MPD and then n-hexane soln of TMC	The results showed improvement of surface properties such as RMS roughness, contact angle and solid–liquid interfacial free energy, a decrease in film thickness and an increase in pore size and water flux	Fathizadeh et al. (2011)
Polyamide(MPD—TMC)	HNT	IP by pouring MPD over PES Support. TMC solutions in cyclohexane then added to the substrate	Shows increase in hydrophilicity, surface roughness and water flux. Higher loading of HNT results in increase in flux, but decrease in salt rejection	Ghanbari et al. (2015)
Polyamide(MPD - TMC)	silicalite-1 nanozeolite	IP carried out between semi-aligned functionalised CNTs over PES support. Immersed in aq MPD and non aq TMC.	Excellent permeability and chemical stability	Huang et al. (2013a)
Polyamide(MPD—TMC)	zwitterion functionalized CNT	IP over PES support. Immersed in aq MPD and then n-hexane soln of TMC	Increased salt rejection and flux	Chan et al. (2013)
Polyamide(MPD—TMC)	Modified carboxy-functionalized MWNT	IP over PES support. Immersed in aq MPD and then n-hexane soln of TMC	Increasing loading showed increase in flux without significant decrease in salt rejection. Improvement in antifouling and antioxidative properties	Zhao et al. (2014)
Polyamide(MPD—TMC)	Reduced graphene oxide/TiO_2_	IP over PES support. Immersed in aq MPD and then n-hexane soln of TMC	Improved water permeability, salt rejection, antifouling property, and chlorine resistance by increasing hydrophilicity, negative surface charge and roughness of PA layer	Safarpour et al. (2015)
Polyamide(PIP-TMC)	PMMA-MWNTs	IP over PES support. Immersed in aq PIP and thentoluenesoln of TMC	—	Shen et al. (2013)
NMP-PES	nano-Fe_3_O_4_	Solution dispersion blending process and PI	—	Alam et al. (2013)
aPES/HBP	HBP-g-sillica	—	enhanced the chlorine resistance of the RO membrane, improved water permeability	Kim K. S. et al. (2013)

### 4.1 Position of Fillers

It is difficult to regulate the position of the filler in TFNs. It has been reported that when NaA nanoparticles are dissolved in the aqueous phase, they are more concentrated near the porous sublayer as compared to near the surface of thin film. On the other hand, when NaA nanoparticles were dissolved in the organic phase, they were found to be homogeneously distributed with equal concentrations near the surface and the sublayer (Huang et al., 2013b). It has been reported that the position of the fillers in the membrane can be tuned by selecting an appropriate type of nanoparticle.

Additive nanomaterials have been demonstrated to improve the performance of TFNs. This has been discussed in the following section.

## 5 New Functionalities Introduced by Additive Nanomaterials

**FIGURE 5:**
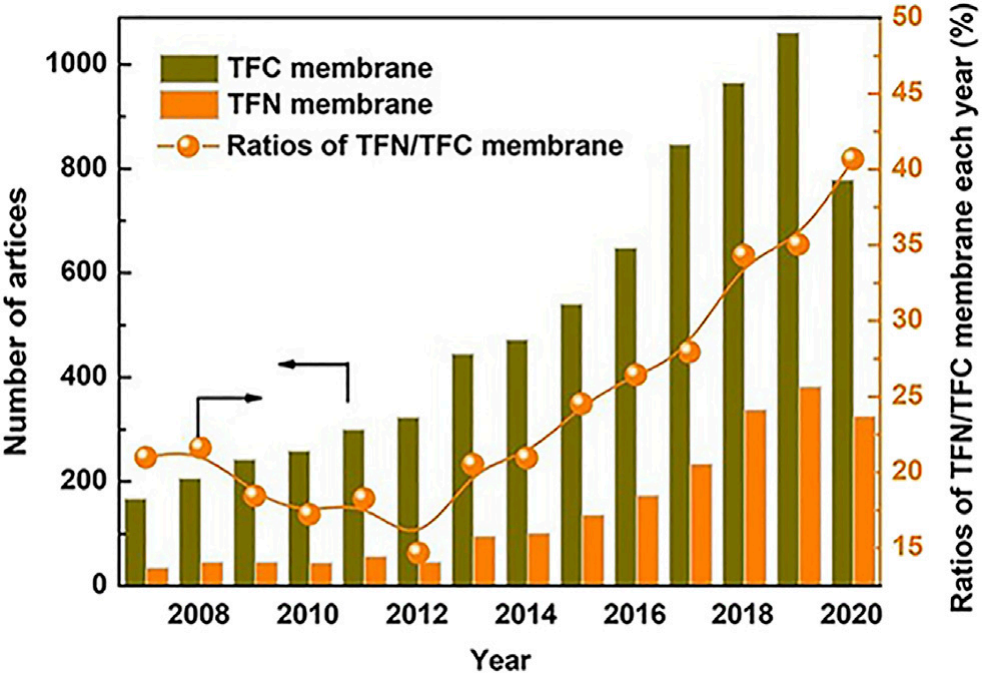
Year wise Publication status of TFNs (Liao et al., 2021).

The addition of nanoparticles (NPs) provides significant improvements to the membrane performance(Liao et al., 2021).Moreover, the year wise publication status for TFN membranes is also shown in Figure 5. The various NPs that are added in order to aid the adsorption, photocatalysis and antimicrobial properties of TFNs have been discussed in the following subsections.

### 5.1 Adsorption

Membranes can be enabled to adsorb heavy metals from water by the incorporation of NPs inside the polymer matrix. Daraei developed a method to remove copper from aqueous solutions by incorporating PANI/Fe_3_O_4_ NPs inside the PES matrix through PI method (Daraei et al., 2012). Elsewhere, Fe-Mn binary oxide (FMBO) is used to remove As (III)] (Jamshidi Gohari et al., 2013). These highlight the possibility of nanocomposite membranes being incorporated with adsorbents for removal of contaminants.

### 5.2 Photocatalysis

TiO_2_ has been known for its photocatalytic properties and as a result has been used for applications such as water splitting, treatment of water and self-cleaning of surfaces. Its stability, ease of preparation and commercial availability add to its functionality (Mills and Le Hunte, 1997; Paz, 2010). Rahimpour found that UV-irradiated TiO_2_/PES nanocomposite membranes had higher flux and improved fouling resistance compared to ordinary nanocomposite membranes, attributing the improvement to the photocatalysis and high hydrophilicity of TiO_2_ under UV irradiation (Rahimpour et al., 2008).

### 5.3 Antimicrobial Activity

Membrane biofouling, which is caused by microbial growth, has been a major challenge to membrane technology (Zhu et al., 2010). It increases energy costs, decreases permeability, and reduces permeate quality. Hence, developing antimicrobial membranes can result in significant enhancement in efficiency of the separation process. Silver (Ag), due to its impressive biocidal properties, is a highly explored antimicrobial agent and has proven applications in avenues such as antimicrobial coatings, plastics, and wound dressings (Liu et al., 2008; Lind et al., 2009b). Addition of Ag_2_O nanoparticles not only acts as biocidal agent but also improves the desalination performance of the membrane (Al-Hobaib et al., 2015). Chou used Ag NPs in CA matrix and Zodrow incorporated Ag NPs into PSU matrix to improve biofouling resistance (Chou et al., 2005; Zodrow et al., 2009).Table 2 provides a summary of the variety of nanoparticles that are used in TFNs (Mills and Le Hunte, 1997; Chou et al., 2005; Liu et al., 2008; Rahimpour et al., 2008; Zodrow et al., 2009; Paz, 2010; Zhu et al., 2010; Daraei et al., 2012; Kim E. S. et al., 2013; Huang S. G. et al., 2013; Jamshidi Gohari et al., 2013; Duan et al., 2015a).The various performance parameters that are utilized in order to characterize TFNs have been discussed in detail in Section 6.

**TABLE 2:** Summary of nanoparticles used in TFNs (Fathizadeh et al., 2011; Daraei et al., 2012; Huang et al., 2013a; Kim E. S. et al., 2013; Alam et al., 2013; Baroña et al., 2013; Huang et al., 2013b; Chan et al., 2013; Shen et al., 2013; Zhao et al., 2014; Ghanbari et al., 2015; Safarpour et al., 2015).

Nanoparticle	Aqueous phase	Salt in feed	Applied pressure(psi)
multiwalled cnt; <8 nm diameter	MPD 2%wt	NaCl 1,000 ppm	100
halogen reactive, nitrogen from amines, imides, sulfonamides	MPD 4%wt	—	—
Zeolite A (0.4% w/v) (in organic phase)	MPD 2%wt	NaCl 2,000 ppm	180
Linde type A(LTA) zeolite nanocrystals	MPD(2–3% w/v) TEACSA(6% w/v) SLS(0.02% w/v) IPA (0–29% w/v)	NaCl 2,000 ppm	224.8
TiO2(in organic phase)	MPD (2%wt) NaOH(0.05wt%)	MgSO_4_ 2,000 ppm	87
Ag nanoparticles	MPD(2%wt) NaOH	MgSO_4_ 2,000 ppm	125–250
0.2 w/w% zeolite-A nanoparticles in the TMC solution	MPD(2% w/v) TEA(2%)CSA(4% w/v) SLS(0.02% w/v) IPA (10% w/v)	NaCl 10 mM soln	225
Polyhedral Oligomeric Silsequioxane(POSS) (in organic phase)	MPD(2wt%)	NaCl 2,000 ppm	225
Oxidized MWNT			
** **AluminoSilicate SWNT	—	NaCl and MgSO_4_ 2,000 ppm each	—
** **nanozeolite NaX	MPD	—	175
** **HNT	MPD 2% w/v	NaCl 2,000 ppm	218
** **silicalite-1 nanozeolites	MPD 2% w/v	—	—
** **zwitterion functionalised CNT	MPD 2% w/v	NaCl 1,000 ppm	530
** **Modified carboxy-functionalised MWNT	MPD 2% w/v	NaCl 2,000 ppm	232
** **Reduced graphene oxide/TiO_2_	MPD 2%wt	NaCl 2,000 ppm	218
** **PMMA-MWNTs	PIP	NaCl 2,000 ppm	145
** **nano Fe_3_O_4_	NMP	—	—
** **mesoporous SiO_2_ nano-particles	sulfonated poly(arylene ether sulfone)	NaCl 2,000 ppm	225
** **HBP-g-silica	aPES-MPDA-TEA	—	—
** **nano ZnO	MPD(2%)-TMC(0.1%)	—	—

## 6 TFN Membrane Performance Characteristics

Salt rejection and water flux are the two most important parameters that are used to judge the performance of TFNs.Some of the most important membrane performance parameters have been discussed in the following subsections. These parameters depend upon many factors, some of which are surface roughness, hydrophilicity, surface charge density (Lu et al., 2013; Safarpour et al., 2015). The performance TFNs can be tailored as required by using different nanoparticles as fillers in the thin film, or by even using different methods to synthesize the membrane (Safarpour et al., 2015). Table 3 lists the different types of nanoparticles used as fillers in TFN membranes reported in literature. For each TFN, the water flux and salt rejection at a particular percentage loading of nanoparticles and the polymer matrix for the membrane is also reported. The last two membranes in Table 3 are exceptions, as they are not synthesized by IP method. Each of the following subsections deals with commonly used performance parameters.

**TABLE 3:** Water flux and NaCl rejection at given loading of nanoparticle fillers in TFN membranes (Fathizadeh et al., 2011; Daraei et al., 2012; Huang et al., 2013a; Kim E. S. et al., 2013; Alam et al., 2013; Baroña et al., 2013; Huang et al., 2013b; Chan et al., 2013; Shen et al., 2013; Zhao et al., 2014; Ghanbari et al., 2015; Safarpour et al., 2015).

Filler	Polymer matrix(for membranes manufactured by IP)	% Loading	Water flux (L/m^2.h)	Salt rejection (%)	Salt solution	Pressure psi
Zeolite particles(NaA)	MPD-TMC	0.4(w/v)	0.95	92.0 ± 1.9	NaCl 2,000 ppm	179
Linde type A-1 (NaA)	MPD-TMC	0.2(w/v)	66.6	92.0 ± 0.5	NaCl 2,000 ppm	224.8
AgA	MPD-TMC	0.4 (w/v)	42.5 ± 1	93.5 ± 1	NaCl 2,000 ppm	225
MWNT (conventional process)	TEOA-TMC	0.05(w/v)	2.6 ± 0.1	Not reported	N/A	87
MWNT (improved process)	TEOA-TMC	0.05(w/v)	4.5 ± 0.5	Not reported	N/A	87
Ag20 (max - flux condition)	MPD-TMC	0.03(wt)	40.43 ± 3	99 ± 0.1	NaCl 2,000 ppm	225
Ag20 (max - rejection condition)	MPD-TMC	0.03(wt)	40.43 ± 3	99 ± 0.1	NaCl 2,000 ppm	225
MgTiO3	MPD-TMC	0.1(wt)	45	98	NaCl 2,000 ppm	225
Al2O3	MPD-TMC	1 wt	5	88	NaCl 2,000 ppm	145
Aluminosilicate single walled nanotubes	MPD-TMC	0.59 wt	24.6	96.24	0.034 M	232
HNT (max flux)	MPD-TMC	0.1(w/v)	48 ± 3	80 ± 3	NaCl 2,000 ppm	218
HNT (max rejection)	MPD-TMC	0.05(w/v)	36 ± 2	95 ± 2	NaCl 2,000 ppm	218
CNT	MPD-TMC	0.1(w/v)	28.05	90	NaCl 2,000 ppm	232
Silica (mcm-41) nanoparticles (max flux)	MPD-TMC	0.05(wt)	46.6	97.9	NaCl 2,000 ppm	290
Silica (mcm-41) nanoparticles (max rejection)	MPD-TMC	0.1(wt)	46	98.9 ± 3	NaCl 2,000 ppm	305
non porous spherical silica nps (max flux)	MPD-TMC	0.1(wt)	36 ± 2	97.6 ± 2	NaCl 2,000 ppm	319
non porous spherical silica nps (max rejection)	MPD-TMC	0.05(wt)	35 ± 2	98.1 ± 2	NaCl 2,000 ppm	334
Zwitter ion functionalised CNTs (max flux)	MPD-TMC	20 wt	28.5	98.6	NaCl 1,000 ppm	530
Carboxy functionalised MWNTs (max flux)	MPD-TMC	0.1(wt)	28 ± 2	90	NaCl 2,000 ppm	232
Reduced graphene oxide/TiO2	MPD-TMC	0.02(wt)	51.3	99.45	NaCl 2,000 ppm	218
PMMA-MWNT	PIP-TMC	0.67 g/L	5	44.1	NaCl 2,000 ppm	145
POSS-1 (max flux)	MPD-TMC	0.4 w/v	33. ± 3	98.2 ± 0.3	NaCl 2,000 ppm	225
POSS-2 (max flux)	MPD-TMC	0.4 w/v	27.1 ± 1.1	98.9 ± 0.2	NaCl 2,000 ppm	225
POSS-3 (max flux)	MPD-TMC	0.4 w/v	33.4 ± 1.1	98.6 ± 0.3	NaCl 2,000 ppm	225
POSS-4 (max flux)	MPD-TMC	0.4 w/v	3.2 ± 0.7	95.9 ± 0.6	NaCl 2,000 ppm	225
ZIF-8 (max flux)	MPD-TMC	0.4 (w/v)	51.92 ± 1.1	98.5 ± 0.3	NaCl 2,000 ppm	225
ZIF-8 (max rejection)	MPD-TMC	0.1 (w/v)	36 ± 1.2	99.2 ± 0.4	NaCl 2,000 ppm	225
acidified MWCNT (max flux)	MPD-TMC	0.1 (w/v)	71	82	NaCl 2,000 ppm	232
acidified MWCNT (max salt rejection)	MPD-TMC	0.1 (w/v)	20	94	NaCl 2,000 ppm	232
Nano-ZnO	MPD-TMC	0.5 wt%	32	98	Not reported	225
nano-Fe3O4 (max flux condition)	PES dissolved in NMP (not by IP)	15(wt)	280 ± 3	39	NaCl 2,000 ppm	145
nano-Fe3O4 (max rejection condition)	PES dissolved in NMP (not by IP)	10(wt)	75 ± 3	68	NaCl 2,000 ppm	145

### 6.1 Water Flux

The measurement of water flux in thin film nanocomposite membranes was performed using a cross-flow membrane module. The water flux was calculated using Equation 1:

F=V/(At), (1)

Where F is the pure water flux, V is total volume of permeated pure water, A is area of the membrane, and t is the operation time (Wu et al., 2010).

Nanoparticles in the thin film membrane can lead to an increase in hydrophilic nature and decrease in cross-linking of the membrane which consequently contributes to high flux of water through the membrane. Increased water flux can also depend upon the structure of the nanoparticle used. For example, MCM-41 silica nanoparticles are porous in nature (Yin et al., 2012). These pores inside the nano-particles present in the thin film membrane can act as short pathways for preferential passage of water molecules through the nanoparticle (Yin et al., 2012). Water tends to move faster through hydrophobic porous particles than hydrophilic non-porous particles (Duan et al., 2015b).The nanomaterials possess high surface porosity resulting in improved salt rejection capacity and reduced macro void formation.

Carbon nanotubes also facilitate high flux of water, as they act like channels for transportation of water (Safarpour et al., 2015). This reduces the water transport route as the water molecules can enter into part of the multiwalled carbon nanotubes (MWCNTs) instead of passing through the entire PA film. Aggregation of these nanoparticles might also lead to the formation of a network inter-connected with other pores in the membrane leading to a higher increase in water flux (Zhang et al., 2011).

### 6.2 Salt Rejection

For all the data reported, salt rejection is calculated using the formula:

$$\text{R}=\;1-{\text{C}}_{\text{p}}/{\text{C}}_{\text{f}},$$ (2)

Where R is salt rejection, C_p_ is concentration of salt in the permeate solution, and C_f_ is concentration of salt in the feed solution (Wu et al., 2010).

Salt rejection is governed by factors such as defects and molecular sieving. Also, it is known that for transport of ions of different valences through carbon channels with negatively charged functional groups, ion exclusion depends more upon electrostatic interactions (Donnan exclusion) rather than steric hindrance (Shen et al., 2013). It is also known that high crosslinking in the MPD-TMC (m-phenylene diamine—trimesoyl chloride) layer causes higher salt rejection and lowerNaClpermeance (Duan et al., 2015b).

As reported by Safarpour, increase in rGo/TiO_2_ loading in thin film membrane leads to decrease in roughness of membrane (Safarpour et al., 2015). This is due to the increase in hydrogen bonding between the hydrophilic nanoparticles and polyamide layer. It was also observed that increase in nanoparticle loading causes decrease in contact angle with water, which is an indication of increasing hydrophilicity of membrane. This can be understood based on the increase of surface charge density. Another trend observed is how the pure water flux increases with the increase in nanoparticle loading. This is expected as an increase in hydrophilicity leads to increased flux (Safarpour et al., 2015).

### 6.3 Selectivity Versus Permeability

Permeability and selectivity are two major performance factors for membrane technology. In improving the performance of membranes, there is always a trade-off between these two factors. In an attempt to achieve high levels of water flux, a decrease in salt rejection is obtained. For example, work done by Jun Yin shows a large trade-off between flux and salt rejection (Yin et al., 2012). Pure water flux increases with percentage loading of MWCNTs, from 20 LMH at zero loading of MWCNTs to 71 LMH at 0.1% w/v of MWCNTs. Salt rejection is observed to decrease with increase in loading of MWCNTs, from 94% at zero loading of MWCNTs to 82% at 0.1% w/v of MWCNTs.

The incorporation of nanomaterials could alter the physicochemical properties of the membrane such as cross linkage, charge density and hydrophilicity. This provides specific water pathways that could conquer the permeability-selectivity trade-off. These facts are evident by the advantage of polydopamine coating that provides ZIF-8 nanoparticles with dispersibility in water (You et al., 2021) and other recent works on TFN membranes (Jeon and Lee, 2020; Saleem and Zaidi, 2020; Siew Khoo et al., 2021). Such TFN membranes exhibited improved permeability in desalination.

Almost all studies that used hydrophilic nanofillers showed a decreased contact angle in the TFNs, proving improved surface hydrophilicity. With increase in zeolite loading from 0 to 0.4% (w/v) the contact angle of zeolite-PA TFN membrane has been seen to decrease from around 70^o^ to 40^o^ (Jeong et al., 2007). Other examples of oxidized MWNTs-PA TFN membranes and silica-PA TFN membranes have also displayed a significant reduction in contact angle with increase in loading up to certain specific values (Zhang et al., 2011; Yin et al., 2012). This indicates enhanced water permeability with an increase in nanofiller loading.

Two possible explanations have been proposed for the decrease in the contact angle due to the nanoparticles (NPs). The first ascribes the increased surface hydrophilicity to the hydrolysis of acyl chloride resulting in the generation of carboxylic acid functional groups. This phenomenon occurs due to increasedcount of surface acyl chloride groups in TMC that remain without reacting with the dimethyl pimelimidate’s (DMP) amine group as a result of NPs hydrating and releasing heat when in contact with the MPD aqueous solution (Kim et al., 2000; Ghosh et al., 2008). The second suggests that the presence of embedded hydrophilic NPs on the membrane surface offers larger number of hydrophilic functional groups to the surface.

Along with hydrophilicity, the thickness and the cross-linkage condition of the thin-film layer are key factors in determining the water permeability and selectivity (Ghosh et al., 2008). Generally, a lesserextent of cross-linking and thinner films offer higher permeability. Incorporation of NPs in the PA matrix could reduce cross-linking in the thin-film layer by disrupting the reaction between amine groups and acyl chloride groups. Lind showed through FTIR and XPS results that the cross lining in all their TFN membranes were less compared to the corresponding TFC membranes (Lind et al., 2010). However, it was put forward that molecular sieving or defects could have played an importantpart in the performance.

Furthermore, the inclusion of NPs may also produce additional channels for the flow of water while excluding the solutes. A higher value of water flux with constant salt rejection in zeolite-A NP TFNs was reported by Jeong(Jeong et al., 2007). The hydrophilic molecular sieving NPs may provide preferential flow paths for water molecules. Yin showed that mesoporous silica NPs with highly ordered hexagonal pores exhibit higher permeability compared to nonporous silica NPs (Yin et al., 2012). The nonporous silica NPs resulted in cross linking thereby increasing permeability compared to regular TFCs. The increase in permeability was even higher for the mesoporous silica NP TFNs.

It is desired to significantly improve permeability while maintaining the same salt rejection. However, in order to exploit the favorable properties of the NPs, it is necessary to optimize the internal structure, size and surface properties while ensuring suitable interfacial interactions with the polymer.

### 6.4 Antifouling

Extensive research has been conducted on incorporating nanoparticles that reduce the fouling of membranes, hence prolonging the duration for which the membrane can be used. It is known that the antifouling ability is related to the hydrophilic nature, negative charge and smoothness of the membrane (Shen et al., 2013). The anti-fouling performance of membranes can be evaluated by conducting filtration experiments. The membrane is first compacted using distilled water. This ensures that an almost constant permeate flux is obtained. This is necessary as the initial flux affects the extent of fouling. A protein solution, for example BSA (bovine serum albumin) that possesses synergistic fouling effects, is then taken in the reservoir in order to conduct the permeating experiment for a definite time period. The fouling capacity is measured based on the decline in the flux with time (Shen et al., 2013).

**FIGURE 6:**
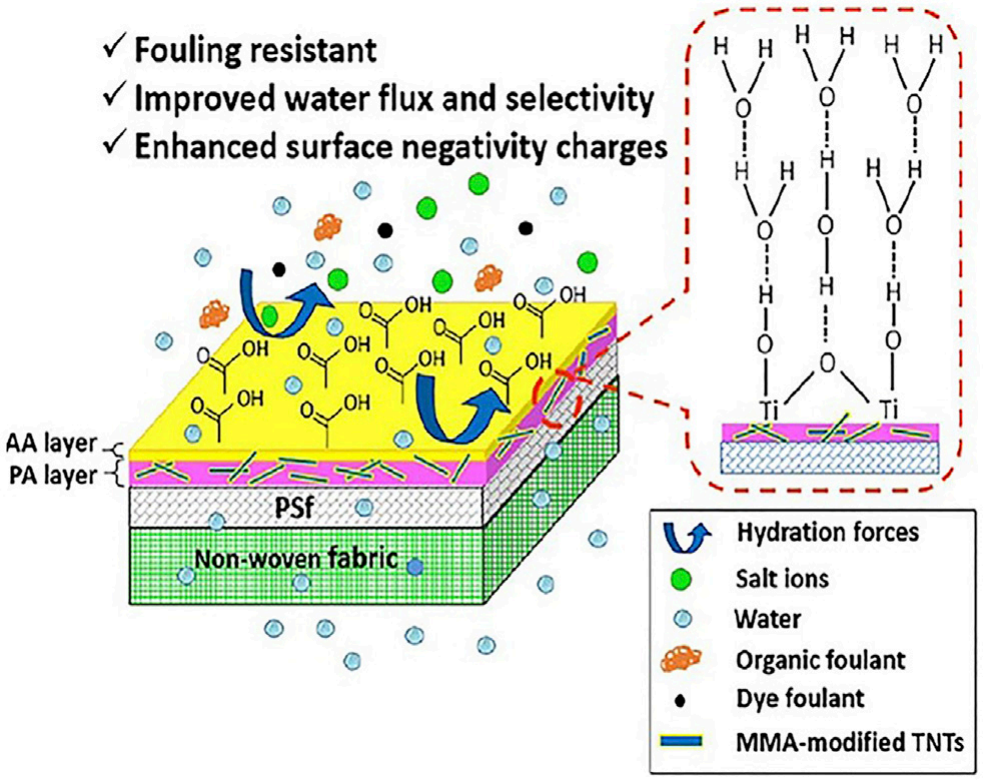
Improved antifouling behaviour through AA-modified TFN membrane (Siew Khoo et al., 2021).

The incorporation of hydrophilic NPs into the PA structure has been shown to increase hydrophilicity of the surface and usually helps diminish surface fouling. Long term fouling tests with silica particles, chloroform, and sodium humate have shown TFN membranes to have a much lower flux reduction when compared to TFC membranes (Rana D. et al., 2011). Extensive research work has been conducted to investigate the antifouling ability of TFNas shown in Figure 6 (Safarpour et al., 2015; Siew Khoo et al., 2021). In order to compare the antifouling properties, water flux through a pristine RO membrane was compared to flux through a TFN with 0.02 % wt. loading of rGo/TiO_2_ under the same applied pressure. The flux through the former dropped to 49% of its value whilst the flux through later decreased to 75% of its value after 180 min of filtration (Safarpour et al., 2015). Hence the presence of fillers in thin film membrane leads to better antifouling resistance. Similarly, recent reports show how the introduction of carboxyl-functionalized MWCNTs reduces fouling (Zhao et al., 2014). This has been attributed to the negative surface charge and greater surface hydrophilicity (Lau et al., 2015).

### 6.5 Chlorine Resistance

Polyamide (PA) based membranes do not have a good resistance to continuous exposure of oxidizing agents. Chlorine is widely used as disinfectant in water treatment (Park et al., 2008). However, chlorine is also a strong oxidizing agent and feed water coming to membrane units from disinfection plants contains chlorine. PA membranes cannot tolerate water having chlorine even in the order of few parts per billion, and the chlorine treatment is required to check the development of biofilms on the membrane. Removing the chlorine from water is an undesired option as this will increase the number of treatment steps, and the overall cost of water treatment. Hence there is a need to increase the resistance of membranes to chlorine (Lau et al., 2015). In order to study the chlorine resistance of a membrane, it is subjected to a high concentration of free chlorine for a short time, which is essentially analogous to the exposure of the membrane for a long time to free chlorine of low concentration (Shen et al., 2013). In order to conduct this study, chlorinate solution is prepared and, the water flux and salt rejection of the required membrane, before and after chlorination is measured and compared (Shen et al., 2013).

As reported by Safarpour et al. (2015), salt rejections before and after exposure to sodium hypochlorite for pristine membrane and TFN were compared to study the chlorine resistance (Safarpour et al., 2015). The bare membrane (without any modifications) showed a decrease in salt rejection from 95.4 to 65.38%, while the TFN membrane with 0.02% loading of rGo/TiO_2_ showed decrease from 99.45 to 96.4% (Safarpour et al., 2015).

Exposure to chlorine can cause ring-chlorination and N-chlorination reactions which disrupt the symmetry of the PA layer converting it from a crystalline state to an amorphous one (Safarpour et al., 2015). This leads to larger free volumes and flexibility of the PA layer that allow salt molecules to pass through easily. On the other hand, the intermolecular hydrogen bonds are amplified by interaction between rGo/TiO_2_ particles and membrane active layer (Safarpour et al., 2015). This can provide obstruction to the substitution of hydrogen with chlorine on the amide groups of the aromatic polyamide membranes resulting in an increase in the chlorine resistance of the membrane. TFNs having carboxyl-functionalized MWCNTs as fillers have also been known to show high chlorine resistance (Park J. et al., 2010). Interactions between the carboxyl group and amide layer is understood to be the reason causing this behavior. Table 4 attempts to summarize a majority of the TFNs reported in literature (Petersen, 1993; Mills and Le Hunte, 1997; Kim et al., 2000; Williams, 2003; Kim et al., 2004; Chou et al., 2005; Wang et al., 2005; Louie et al., 2006; Jeong et al., 2007; Lee et al., 2007; Ghosh et al., 2008; Lee et al., 2008; Liu et al., 2008; Park et al., 2008; Rahimpour et al., 2008; Singh and Aswal, 2008; Ghosh and Hoek, 2009; Jadav and Singh, 2009; Lind et al., 2009b; Zhou et al., 2009; Zodrow et al., 2009; Jadav et al., 2010; Li and Wang, 2010; Lind et al., 2010; Ma et al., 2010; Park K. T. et al., 2010; Park J. et al., 2010; Paz, 2010; Wu et al., 2010; Zhu et al., 2010; Rana D. et al., 2011; Rana T. M. D. et al., 2011; Roy et al., 2011; Kim and Deng, 2011; Kong et al., 2011; Pendergast and Hoek, 2011; Zhang et al., 2011; Baroña et al., 2012; Daraei et al., 2012; Kim et al., 2012; Lau et al., 2012; Ma et al., 2012; Saleh and Gupta, 2012; Yin et al., 2012; Zhao et al., 2012; Alam et al., 2013; Amini et al., 2013; Bao et al., 2013; Baroña et al., 2013; Chan et al., 2013; Daraei et al., 2013; de Lannoy et al., 2013; Hu et al., 2013; Huang et al., 2013a; Huang et al., 2013b; Jamshidi Gohari et al., 2013; Kim S. G. et al., 2013; Kim E. S. et al., 2013; Kim S. G. et al., 2013; Li and Wang, 2013; Lu et al., 2013; Li et al., 2014; Pendergast et al., 2013; Rajaeian et al., 2013; Shen et al., 2013; Wu et al., 2013b; You et al., 2013; Yu et al., 2013; Wu et al., 2013a; Hermans et al., 2014; Subramani et al., 2014; Zhao et al., 2014; Al-Hobaib et al., 2015; Duan et al., 2015a; Duan et al., 2015b; Ghanbari et al., 2015; Hermans et al., 2015; Lau et al., 2015; Safarpour et al., 2015; Shenvi et al., 2015; NanoH2O and Inc. InterNano, 2021). The arrows under the application column denote the performance of the TFN membrane as compared to the parent TFC membrane. For example, in the first row, the fouling resistance of the membrane with Oxidized MWNT nano-fillers increases when compared with the fouling resistance of the TFC membrane without the nano-fillers.

**TABLE 4:** Summary of TFNs and their composition, performance, fabrication and applications.

TFN		Particle size	Loading wt%(best performance)	Fabrication method	Application	Performance	References
Filler	Polymer						
Oxidized MWNTs	Pebax 1074 or PVA	OD:20–40 nm; L:5–15 µm	0–20% of polymer(10% of PVA)	Coating + solvent evaporation	Water/oil emulsion separation	Under 100 psi, optimal water flux is 330 L/m2h, organic solute rejection is 99.8%; Fouling resistance ↑	Wang et al. (2005)
Zeolite(NaA)	PA	50–150 nm	0.004–0.4% (w/v) in organic phase	IP	RO	Surface hydrophilicity ↑; Pw ↑; Salt rejection no change; New concept: TFN	Jeong et al. (2007)
Ag NPs	PA	50–100 nm	10% of polymer in organic phase	IP	NF	Water flux and salt rejection no change; Good antibiofouling property	Lee et al. (2007)
TiO2 (P25)	PA	30 nm	1.0–9.0% (5.0%) organic phase	IP	NF	Under 87 psi, optimal water flux is 9.1 L/m2h, MgSO_4_ rejection (95%, 2000 mg/L)	Lee et al. (2008)
Silica(LUDOX HS-40)	PA	13.2 nm	5–28% of PA	IP	Dioxane solution filtration	Pw ↑; Solute rejection ↓	Singh and Aswal, (2008)
Zeolite (NaA and AgA)	PA	50–250 nm	0.4% (w/v) in organic phase	IP	RO	Pw ↑; Salt rejection no change; AgA-TFN membranes exhibited more hydrophilic and smooth surfaces	Duan et al. (2015a)
Zeolite	PA	97, 212–286 nm	0.2% (w/v) in organic phase	IP	RO	Smaller NPs produced higher permeability enhancements, but larger NPs produced more surface properties change	Li et al. (2013)
Silica	PA	3–16 nm	0–0.4% (3 nm) and 0–0.5% (16 nm) in aqueous phase	IP	RO	Pw ↑; NaCl rejection ↗↘; Thermal stability ↑	Jadav and Singh, (2009)
Oxidized MWNT’s	PVA	OD:8–15 nm; L:10–50 µm	10% of PVA	Coating + Cross-linking	UF of oil/water emulsion	Pw ↑; Solute rejection slightly decreased; Suggested the presence of directional water channels through the interface between filler and PVA matrix	Ma et al. (2010)
Cellulose Nanofibers		OD: 5 nm; L < 10 µm	0.25 and 1.25% of PVA				
Carboxylic MWNTs	Polyester	OD < 8 nm; L = 10–30 µm	0.05%(w/v) in aqueous phase	Modified IP; O/A/O	NF	Pw ↑; Na_2_SO_4_ rejection ↑; Immerse support layer into organic phase before conventional IP process improved TFN performance	Wu et al. (2010)
MWNTs	PA	OD = 9–12 nm; L = 10–15 μm	0.1, 0.5, 1, 5% (w/v) in aqueous phase	IP	RO	Surfactant (Triton X-100) was used to facilitate the dispersion of MWNTs; Chlorine resistance ↑	Park J. et al. (2010)
Zeolite (LTA)	PA	∼250 nm	0.2% in organic phase	IP	Seawater RO	Under 800 psi, optimal permeate flux is around 42 L/m2h, NaCl rejection (99.4%, 32,000 mg/L); Defects and molecular-sieving largely govern transport through zeolite-TFN membrane	Lind et al. (2010)
Functionalized Silica	PA	—	0.04, 0.4% in aqueous phase	IP	RO; PV	Small-angle neutron scattering (SANS) was used to study the dispersion of silica NPs in thin-film layer; Thermal stability ↑; Pw ↑; NaCl rejection ↓	Jadav et al. (2010)
Functionalized MWNTs	PA	OD = ∼ 30 nm; L1 = 10–30 μm; L2 = 0.5–2.0 μm	0.01–0.06% in aqueous or organic phase	IP	NF	Pw ↑; Solute rejection no change; Nanogaps around the external surface of fillers provide a low resistance solvent pathway	Roy et al. (2011)
Oxidized; MWNTs	PA	—	0–0.2% (w/v) in aqueous phase	IP	RO	Surface hydrophilicity ↑; Pw ↑; NaCl rejection ↓	Zhang et al. (2011)
Metal alkoxide (TTIP, BTESE, PhTES)	PA	—	0–5% in organic phase	IP	NF/RO	Pore size ↑; Pw ↑; With PhTES, Pw ↑, NaCl rejection no change	Kong et al. (2011)
Zeolite (NaX)	PA	40–150 nm	0.004, 0.01, 0.04, 0.2% (w/v) in organic phase (0.2%)	IP	RO	Thermal stability ↑; Hydrophilicity ↑; Pw ↑; NaCl rejection no change	Fathizadeh et al. (2011)
Hydrophilized ordered mesoporous carbon (OMC)	PA	—	0–10% in aqueous phase (5%)	IP	NF	Hydrophilicity ↑; Protein adsorption ↓; Pw ↑; NaCl rejection ↓; Na2SO4 rejection slightly ↓	Kim and Deng, (2011)
Hydrophilic macromolecules + Ag+	PA	11,000 Da	0.25% of (MDI + PEG) in organic phase; 0.25% of AgNO_3_ in aqueous phase	IP	Seawater RO	Good seawater desalination performance; Fouling resistance ↑; Biofouling resistance ↑	Rana D. et al. (2011)
Ag NPs	PA	Several nanometers	Dispersed in aqueous phase Finally, 10% in PA	IP	NF	Surface hydrophilicity ↑; Pw ↑; Salt rejection no change; Biofouling resistance ↑	Kim et al. (2012)
Mesoporous silica (MCM-41) and nonporous silica	PA	∼100 nm; ∼ 100 nm	0–0.1% in organic phase (0.05%)	IP	RO	Surface hydrophilicity ↑; Pw ↑; Salt rejection no change; Under 300 psi, optimal permeate flux is 46.6 L/m2h, NaCl rejection (97.9, 2000 mg/L); Porous structures of filler contributed significantly to the water flux enhancement	Yin et al. (2012)
Proteoliposome with aquaporin	PA	<150 nm	10 mg/ml in aqueous phase	IP	RO	Pw ↑; Salt rejection no change; Under 72.5 psi, water flux is 20 L/m2h, NaCl rejection (∼97%, 584.4 mg/L)	Zhao et al. (2012)
Aluminosilicate SWNTs	PVA	OD = 2.7 nm; L > 200 nm	0–20% (v/v) in PVA solution	Coating + Cross-linking	NF	Surface hydrophilicity ↑; Roughness ↓; Pw ↑; Salt rejection ↑	Baroña et al. (2012)
Zeolite (NaY)	PA	40–150 nm	0–0.4% (w/v) in organic phase (0.1%)	IP	FO	Pw↗↘; NaCl rejection ↘↗; Surface roughness ↗↘	Ma et al. (2012)
Alumina NPs	PA	∼14 nm	1% in organic phase	IP	NF	Surface hydrophilicity ↑; Pw ↑; Salt rejection no change	Saleh and Gupta, (2012)
Oxidized MWNTs	PA	OD = 5–10 nm; L = 10–30 μm	5% of PA	IP	Oil sand process-affected water treatment	Water flux ↑; Organic fraction rejection ↑; Fouling resistance ↑	Kim S. G. et al. (2013)
Zwitterion functionalized CNTs	PA	OD = 1.5 nm; L = 1 μm	0, 9, 20% of PA (20%)	Deposition + IP	RO	Water flux and salt rejection ↑; Under 530 psi, optimal water flux is 48.8 L/m2h, NaCl rejection (98.6%, 2,542 mg/L)	Chan et al. (2013)
Carboxylic MWNTs	PA	OD < 8 nm; L = 10–30 μm	3 mg per membrane sample	Deposition + IP	RO	High electrical conductivity (∼400 S/m), NaCl rejection (>95%, 1000 mg/L), high water flux; Biofouling resistance ↑ under electric potential	de Lannoy et al. (2013)
Oxidized MWNTs	PVA	OD = 10–30 nm; L = 0.5–2 μm	0, 5, 10, 15% of PVA	Electrospinning + Cross-linking	UF	Water flux ↑; Organic fraction rejection (99.5%); Good mechanical properties	You et al. (2013)
PMMA modified MWNTs	PA	OD = 20–30 nm; L < 50 μm	0–5.4 g/L in organic phase (0.67 g/L)	IP	NF	Pw and selectivity ↑	Shen et al. (2013)
PMMA modified MWNTs	PA	OD = 20–30 nm; L < 10 μm	0.67, 1.33, 2.0 g/L in organic phase (0.67)	IP	NF	Under 145 psi, optimal water flux is 69.7 L/m2h, Na_2_SO_4_ rejection (99.0%, 2000 mg/L)	Yu et al. (2013)
Carboxylic MWNTs	PA	OD < 8 nm; L = 10–30 μm	0–2.0 mg/ml in aqueous phase (0.5)	Modified IP; O/A/O	NF	Pw↗↘; Hydrophilicity ↗↘; Under 87 psi, optimal water flux is 21.2 L/m2h, Na_2_SO_4_ rejection (>70%, 5 mmol/L)	Wu et al. (2013a)
Amine functionalized MWNTs	PA	OD = ∼ 5 nm; L = ∼ 50 μm	0.01, 0.05, 0.1% in aqueous phase	IP	FO	Hydrophilicity ↑; S value ↓; Pw and salt rejection ↑ in both AL-FS and AL-DS modes	Amini et al. (2013)
Zeolite (Silicalite-1)	PA	—	0–0.2% in organic phase	IP	RO	Pw, hydrophilicity, and acid stability ↑; Silicalite-1 is superior to NaA in fabricating TFN	Huang et al. (2013a)
Zeolite (NaA)	PA	—	0–0.2%(w/v) in organic phase	IP	RO	Water flux and salt rejection ↑	Huang et al. (2013b)
Aminated Zeolite	PA	≤ 100 nm	0.02% in aqueous solution	IP	RO	Pw ↑; Chlorine resistance ↑; Under 800 psi, water flux is 37.8 L/m2h, NaCl rejection is 98.8% (32,000 mg/L)	Kim S. G. et al. (2013)
Zeolite A	PA	250 nm	0.2% in organic phase	IP	RO	Pw and salt rejection ↑; Resistance to physical compaction ↑	Pendergast et al. (2013)
Modified mesoporous silica	PA	∼100 nm	0–0.07% in aqueous phase (0.03%)	IP	NF	Under 87 psi, optimal water flux is 32.4 L/m2h, Na_2_SO_4_ rejection (> 80%, 5 mmol/L)	Wu et al. (2013b)
Mesoporous silica	PA	∼164 nm	0–0.1% (w/v) in organic phase (0.1)	IP	RO	Pw and hydrophilicity ↑; Under 232 psi, optimal water flux is 53 L/m2h, NaCl rejection (>96%, 2,000 mg/L)	Bao et al. (2013)
Aminated hyper branched silica	PA	∼7 nm	0.02% in aqueous solution	IP	RO	Pw ↑; Chlorine resistance ↑; Under 800 psi, water flux is 34.5 L/m2h, NaCl rejection is 97.7% (32,000 mg/L)	Kim K. S. et al. (2013)
Silica	Fluoropolyamide	—	0–1.0% (w/v) in aqueous phase (0.1)	IP	NF	Pw ↑; Na_2_SO_4_ rejection ↗↘; Under 87 psi, optimal water flux is 15.2 L/m2h, Na_2_SO_4_ rejection (85.0%, 2000 mg/L)	Hu et al. (2013)
Aluminosilicate SWNT	PA	OD = ∼ 2.7 nm; L = 150 nm	0.05, 0.1, 0.2% (w/v) in organic phase	IP (single pass flow)	Low pressure RO	Pw and salt rejection ↑; Resistance to physical compaction ↑	Baroña et al. (2013)
Aminosilanized TiO_2_	PA	∼21 nm	0.005, 0.05, 0.1% in aqueous solution (0.005%)	IP	NF	Pw and selectivity ↑; Thermal stability ↑; Under 110 psi, optimal water flux is 12.3 L/m2h, NaCl rejection is 54% (2,000 mg/L)	Rajaeian et al. (2013)
Organoclay (Cloisite 15A and 30B)	Chitosan	—	0.5, 1, 2% in casting solution	Coating on PVDF substrate	NF for dye removal	Dye removal ↑; Adsoption is the dominating removal mechanism	Daraei et al. (2013)
Proteoliposome containing Aquaporin Z	PEI	∼ 107.8 nm	0, 50, 200, 400 in Lipid-to-protein ratio (200)	PEI crosslinking	NF	Under 14.5 psi, optimal water flux is 36.6 L/m2h MgCl_2_ rejection (95%, 100 mg/L)	Li et al. (2014)
Carboxylic MWNTs	PA	OD = 20–40 nm; L = 1–5 μm	0–0.1% in MPD solution	IP	RO	Hydrophilicity ↑; Water flux ↑; Solute rejection no change; Better antifouling and antioxidative properties	Zhao et al. (2014)

IP, interfacial polymerization; PA:polyamide; PV, pervaporation; Pw, water permeability.

The following section discusses various desalination processes and the use of TFNs along with specific modifications to improve the desalination performance of the same in detail.

## 7 Desalination

It is estimated that only 0.8% of the water on Earth is freshwater, while the seas and oceans constitute almost 96% of the water on Earth (Greenlee et al., 2009). To address the increasingly troublesome problem of water shortage around the world, it is important to develop desalination technologies to make use of the salty water from oceans and groundwater aquifers.

The feed water for desalination plants ranges from 10,000 ppm TDS to 60,000 ppm TDS (Mickley, 2001). The concentration of the feed water is the basis upon which desalination plants are designed. Desalination process, energy costs, product recovery and waste management are some of the design choices that are made feed water in mind (Greenlee et al., 2009).

Desalination can be broadly classified into two basic methods; thermal processes and membrane processes (Greenlee et al., 2009). Thermal processes include MED and MFD. MED involves heat transfer between feed water and steam over multiple stages with the aim to desalinate water (Kesieme et al., 2013). The process is optimized with the aim to produce the highest amount of fresh water with the least input of energy. MSF is a commonly used technique which involves passing the feed water through a series of flash chambers to heat the feed seawater, after which the condensate is collected separately (Van der Bruggen and Vandecasteele, 2002). Even though MSF is easier and more reliable than MED, it is more expensive and energy-intensive (Sagle and Freeman, 2004).

Electrodialysis is a type of membrane process, where the water is passed through a series of parallel cationic and anionic membranes, and an electric current is passed through the seawater to cause separation. This method is suitable only for waters having low concentrations, like brackish water (Reahl, 2004). NF is another membrane based process, but cannot be used as a treatment step on its own as it is not able to bring the water down to drinking water standards (Bohdziewicz et al., 1999). Therefore NF is used in association with RO. RO is one of the most popular membrane based processes, achieving salt rejections of greater than 99% (Bates and Cuozzo, 2000). RO membranes can be used for both seawater and brackish waters (Greenlee et al., 2009).

The other methods of desalination are Capacitive Deionization (CDI) andMVC (Bates and Cuozzo, 2000; Van der Bruggen and Vandecasteele, 2002). CDI is an electrochemical method in which the ions are separated by electrosorption onto a porous charged electrode (Zhao, 2013). While CDI has advantages like easy cleaning, low cost and good mechanical properties, there is not enough data for implementation on a large scale (Zhao, 2013). MVC follows the same steps as MED or MFD, except that the vapor is condensed into water using mechanical methods. The energy produced by this is then in turn used to heat the feed. Although this process has a high efficiency, it has some drawbacks like that it is difficult to control and complex and so it is used only in small-scale plants (Sagle and Freeman, 2004).

The following subsections compared the major desalination techniques based on factors like energy demand and cost.

### 7.1 Energy Demands

Energy demand is one of the most important considerations that need to be taken into account for desalination.RO consumes the least amount of energy among MED, MSF and RO (Wade, 1993; Wade, 2001). For RO membranes, energy is consumed in pumping water across the membranes while in MSF and MED energy is consumed in converting water to steam and for running pumps (Sagle and Freeman, 2004). Energy consumption is also affected by the salinity of the feed water (Ettouney et al., 2002). Higher concentration of salt in feed water leads to larger osmotic pressure. Larger osmotic pressure means a larger *trans*-membrane pressure needs to be applied which in turn means larger pressure needs to be applied by the pumps, leading to an increase in the energy consumption (Sagle and Freeman, 2004). The energy consumption of TFNs are improved compared to TFCs for the separation process requirement (Subramani et al., 2014).

**FIGURE 7:**
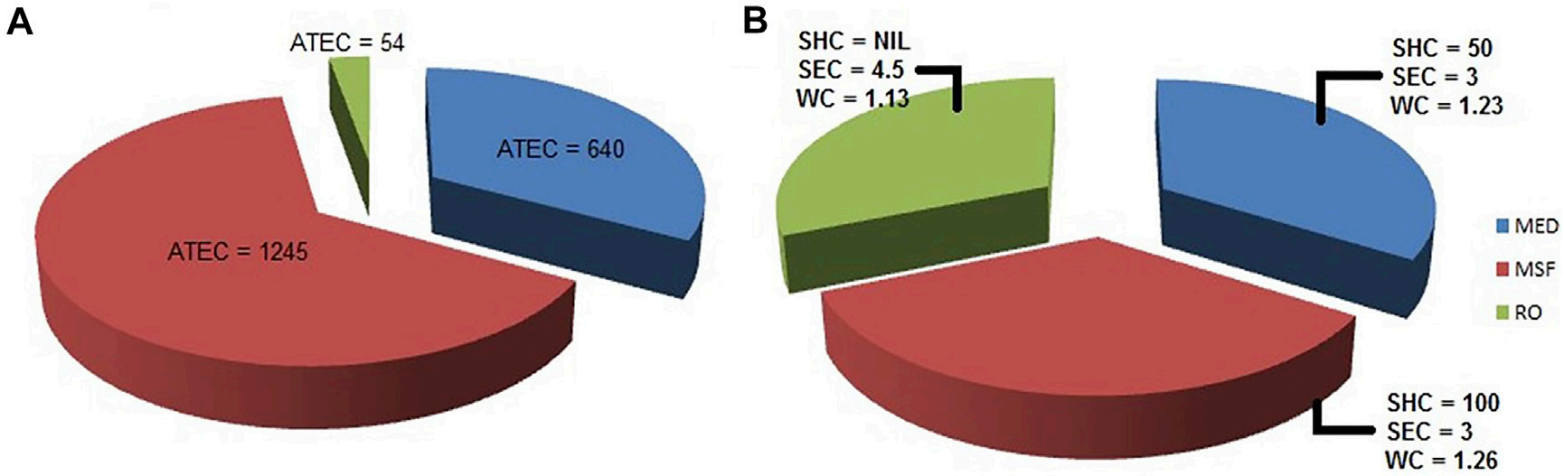
Comparison of MED, MSF and RO. **(A)** Average total energy consumption (ATEC) expressed in MW **(B)** Specific heat consumption (SHC) expressed in kW _th_ h/m³, specific electricity consumption (SEC) expressed in kW _e_ h/m³, and water cost (WC) involved expressed in $1.5/m³.

While MED and MSF require thermal and electrical energy, RO requires only electrical energy. Assuming water production of 290,000 m^3^/day, average total energy consumption (ATEC) in MW (Mega Watt) for the three processes is graphically shown in Figure 7A.

### 7.2 Cost

Figure 7B allows us to compare the three desalination technologies: MED, MSF and RO, based on the energy consumption and the water cost.The energy cost calculations are based on a plant capacity of a volume 32,000 m^3^/day; and a total dissolved solids (TDS) concentration of 42,000 mg/L. The energy cost used is $0.053/kWh. The specific heat consumption (abbreviated as SHC in the figure) is expressed in kW_th_ h/m^3^ and the specific electricity consumption (abbreviated as SEC in the figure) is expressed in kW_e_ h/m^3^. The water cost (abbreviated as WC in the figure) calculations are based on a plant capacity of volume 31,822 m^3^/day and a TDS concentration of 37,000 mg/L. Energy costs are expressed in $1.5/m^3^ (Sagle and Freeman, 2004).

It can be seen that RO has the lowest cost per unit volume. RO membranes have a higher initial cost, but the end result is a higher production rate as compared to MED and MSF (Ettouney et al., 2002; Sagle and Freeman, 2004; Kesieme et al., 2013). In fact, it has been concluded by Kesieme that even with the inclusion of carbon tax in Australia, RO continues to be the most cost effective of the discussed desalination processes (Kesieme et al., 2013).

**FIGURE 8:**
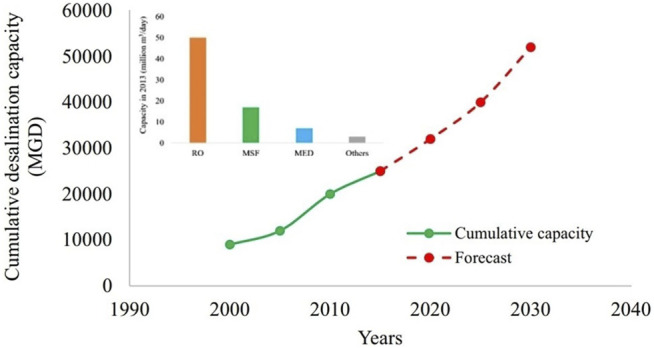
Global cumulative desalination capacity trends and forecast up to 2030 (Zhao et al., 2020).

RO is the predominant technology used for desalination. Figure 8 shows the (global) cumulative desalination capacity trends (Zhao et al., 2020) and forecast up to 2030 (Shahzad et al., 2019).It is a common practice to improve parameters like salt rejection and permeate flux using various kinds of NPs. The following subsection discusses such modifications that have been reported in the recent times in detail.

### 7.3 Improvement in Desalination Performance of TFNs

It is possible to improve the desalination performance of TFNs by incorporating NPs like functionalized silica nanoparticles (SNs) into the membrane. SNs can be synthesized in various sizes and with differing surface functionalities like epoxy, amine and hydroxyl. The chemistry, surface hydrophilicity and morphology of TFNs are affected by the various factors like size, concentration and surface functionality of the used SNs resulting in an increase in the permeate flux without any drastic change in the salt rejection. Similarly, it is possible to incorporate various other kinds of NPs in order to improve the desalination performance of TFNs (Zargar et al., 2017).

A polyamide TFN has also been reported that has been incorporated with multiwalled carbon nanotubes-titania nanotubes hybrids (MWCNT-TN). The use of acid treated MWCNT-TN as a filler in the PA membrane results in improvement in the surface properties of the membrane (surface charge, contact angle and roughness) resulting in an increase in the water permeability with negligible change in the salt rejection (Wan Azelee et al., 2017).

ZnO nanostructures like nanorods (R-ZnO), nanoflowers (F-ZnO) and nanospheres (S-ZnO) have been shown to improve the hydrophilicity of the TFN membrane with increase in the ZnO loading. Amongst three nanostructures, S-ZnO has the largest surface area and smaller size. TFNs with S-ZnO incorporated in them were seen to possess the highest permeate flux with good salt rejection, out of three types of ZnO nanostructures (Rajakumaran et al., 2020).

Similarly, Na + functionalized carbon quantum dot (Na-CQD) incorporated into the PA layer, resulting in a TFN hollow fiber membrane has been shown to possess a much larger effective surface area, a larger number of hydrophilic O-containing groups in the PA layer and a less thickness of the PA layer. These results in a better permeate flux of water enabling use in the desalination of brackish water (Gai et al., 2019).

Another research work done on amino-phenyl modified mesoporous silica NP based TFNs reported a 21.6% increase in water permeability with a marginal decrease in salt rejection (0.29%) compared to pure PA membrane under optimum AMSN dosage of 0.25 g/L (Wang et al., 2019).

TFC membranes modified with Cu-Al layered double hydroxide nanofillers were found to be less negative than the usual TFC membranes and a substantial improvement in the anti-fouling properties was observed indicated by an improvement in water flux by 14% (Tajuddin et al., 2019).

Biopolymer based nanocomposite films, which consist of single dimensional palygorskite (PAL) nanorods and double dimensional montmorillonite (MMT) nanoplatelets in the sodium alginate (SA) film resulted in an enhancement in the tensile stress and the capacity of water resistance of the film (Huang et al., 2018).

**FIGURE 9:**
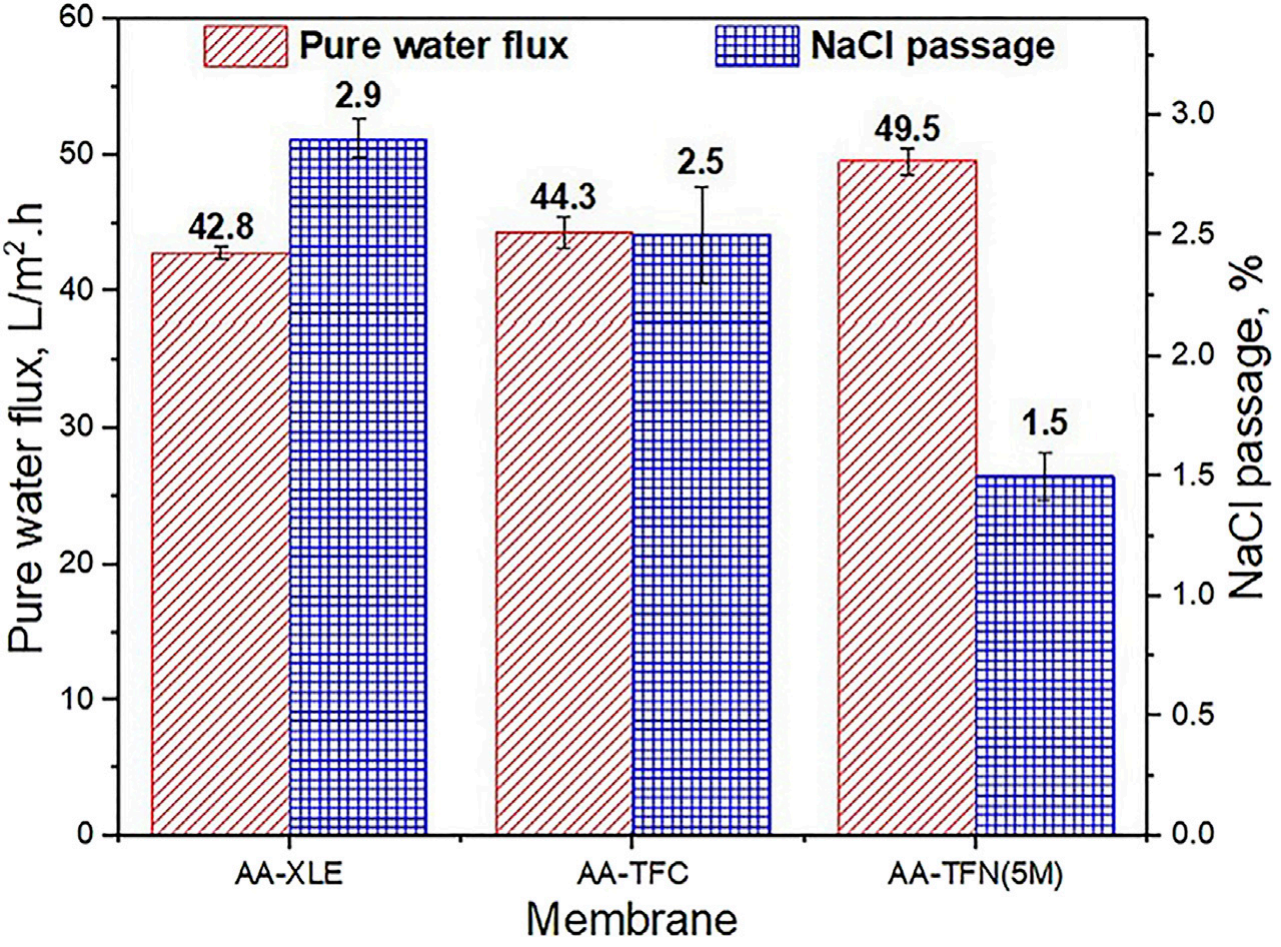
Pure water flux and NaCl permeability without and with acrylic acid (AA) monomer on the PA surface of TFN membrane (Siew Khoo et al., 2021).

All of these above examples showcase some of the numerous recently developed mechanisms to enhance the applicability of thin film nanocomposites in desalination applications by improving the various properties like water permeability and salt rejection of the membrane(Figure 9).However, TFNs continue to have some limitations, especially those concerning the adverse effects of desalination processes on the environment. Some of these concerns have been discussed in Section 7.4.

### 7.4 Environmental Concerns

Thermal emissions, brine discharge and chemicals used in the process all contribute towards environmental pollution (Van der Bruggen and Vandecasteele, 2002). Thermal emissions are directly related to the electric power consumption in the process. Therefore, RO membranes show the least amount of thermal emissions (Van der Bruggen and Vandecasteele, 2002). The impact of brine discharge on the environment can be determined by the temperature and salinity of the waste stream (Van der Bruggen and Vandecasteele, 2002). Brine released at high temperatures in fresh water bodies can decrease the oxygen content of the water hence harming the microorganisms present in the water. Since RO process does not involve heating up the water like in MED or MSF, it causes less harm to the environment in this aspect. When evaluating the environmental impact, all chemical additives that have been added to the water have to be considered (Van der Bruggen and Vandecasteele, 2002). Chemicals that might be used to reduce fouling or scaling, enter fresh water bodies, causing water pollution (Van der Bruggen and Vandecasteele, 2002). Taking all factors into account, it can be seen that RO is the least destructive to surroundings (Sagle and Freeman, 2004).

However, as seen in the previous section, RO involves water wastage. In addition, the demineralized water obtained is unhealthy and the process requires energy input. RO also tends to make the water acidic and is unable to remove chlorine, chloramines, volatile organic compounds (VOCs) and pharmaceuticals. In fact, all conventional methods of water treatment have drawbacks. Ultrafiltration is incapable of removing dissolved inorganic components and involves high energy costs. In microfiltration, fouling is a very serious issue and requires regular cleaning. Similarly, in nanofiltration, membrane fouling results in these membranes having a very limited lifetime. Pre-treatment is often a necessity in the case of nanofiltration (Das et al., 2014).

It has been suggested that CNT membranes possess various favorable properties like anti-fouling function, low energy consumption and self-cleaning functions (Das et al., 2014). This makes them a good alternative to conventional water treatment technologies and they might help in eradicating the fresh water crisis very soon (Das et al., 2014).In the following Section 7.5, the future scope of thin film nanocomposite membrane and their requisite for design and development towards commercialization for various applications is elucidated.

### 7.5 Future Perspective

**FIGURE 10:**
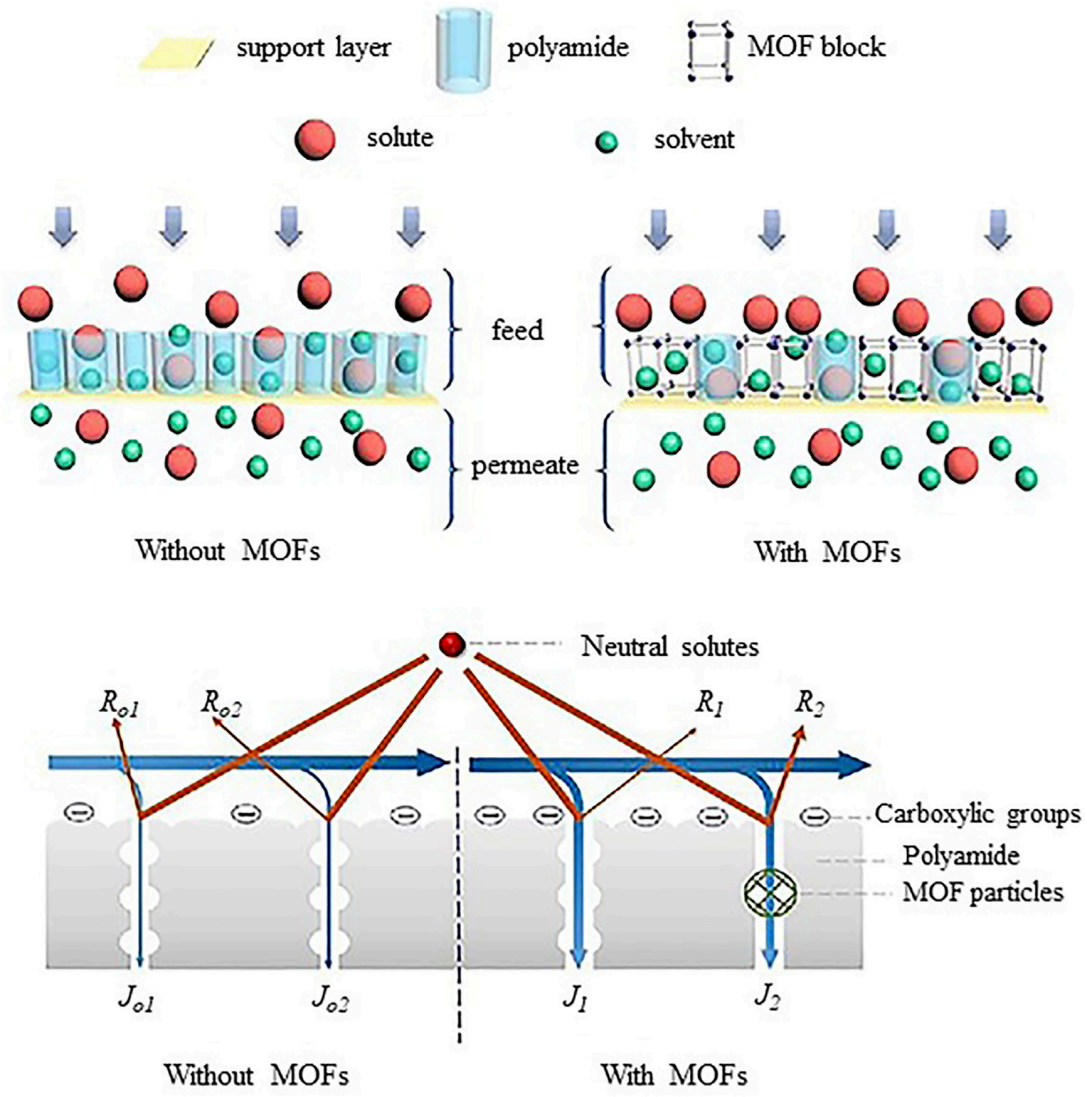
Separation of solute by TFN membranes with and without MOFs (Liao et al., 2021).

Hoek introduced thin film nanocomposite (TFN) RO membranes by incorporating nanoparticles in polyamide layer in 2007 (Jeong et al., 2007). Since then, the new nanoparticles and nanocomposites were researched. The TFN membranes exhibited greater potential in overcoming trade-off between permeability and selectivity. The TFN membranes provided improvement in chlorine resistance and antifouling properties (wang et al., 2011).Despite such unique properties, dispersion of hydrophilic nanoparticles and leaching of nanoparticles into retentate and permeate has raised the environmental concerns and need further research. As the world is facing shortage of freshwater, thin film nanocomposite (TFN) membranes are anticipated to accelerate desalination industry and it can be extended as selective membranes for CO_2_ separation (Wong et al., 2016).The incorporation of functionalized fillers such as GO, CNT, TiO_2_, Ag-TiO_2_, MOFs **(** Figure 10 **)** or organic fillers for specific applications has the potential to enhance membrane performance (Kumar et al., 2020).Recently, an interlayer of nanomaterials (TFNi) membranes showed extraordinary improvement in water flux and selectivity that can be used for the removal of heavy metals and micropollutants at a large scale (Yang et al., 2020).In the near future, high impact TFN membranes with antifouling and biofouling properties, chemical resistance, improved mechanical strength and thermal stability need to be produced by either predisposition of new types of functionalized nanofillers or organic fillers compatible with selective layers of respective membranes. Such design and development of TFN membranes may pave way for more robust membrane systems with increased performance and long term durability providing precise solutions for various commercial water treatment challenges.

## 8 Conclusion

The utilization of nanotechnology in different environments has upgraded the present day environmental engineering and science together with a fresh set of technology that emerged from nanotechnology. The emerged technology at nanoscale has stimulated the advanced utilization of innovative and low-cost techniques that are effective for separation techniques. TFNs are obtained as modifications of TFCs. This modification is in the form of nanoparticles being incorporated into a thin polyamide (PA) dense layer at the top of the TFC membrane, aimed at improving its performance. In particular for desalination, specific modifications are generally made such that the permeate flux increases with negligible changes in the salt rejection upon nanomaterials incorporation. The full potential of a material development is on its performance and feasibility. Both these parameters are being answered in the PA nanocomposites development. The TFNs are used diversely in all membrane based desalination techniques. The transport properties of the penetrants are highly modified with addition of nanoparticles. The functionality of the membrane surface is targeted on specific membrane foulants. A specific kind of requirement is always being supported with a nanocomposites preparation. One of the main limitations of the TFNs is the amount of loading of nanomaterials and effective means for its distribution and dispersion on the whole polymer matrix. The liberty to functionalize the nanomaterials using several chemical groups could also improve their homogenization inside the polymer matrix. It appears that the choice of membrane materials for future RO processes would depend largely on the desired permselectivity and the targeted foulants. This review has focused on a variety TFNs preparedand their enhanced properties for addressing specified desalination requirements. The feasibility of such robust technology specifically meeting environmental impact and energy demands is widely discussed in this review paving way for the progress in research towards the development of new thin film nanocomposite membrane synthesis and manufacturing methods for desalination applications.
